# Unmet Need to Verify Coronary Artery Spasm in Patients with Chronic or Acute Coronary Syndrome and Non-Obstructive Coronary Arteries

**DOI:** 10.3390/life16030412

**Published:** 2026-03-03

**Authors:** Ming-Jui Hung, Ming-Yow Hung

**Affiliations:** 1 Section of Cardiovascular Image, Division of Cardiology, Department of Internal Medicine, Chang Gung Memorial Hospital at Keelung, Chang Gung University College of Medicine, Keelung 204201, Taiwan; 2 Division of Cardiology, Department of Internal Medicine, Shuang Ho Hospital, Taipei Medical University, New Taipei City 23561, Taiwan; 3 Division of Cardiology, Department of Internal Medicine, School of Medicine, College of Medicine, Taipei Medical University, Taipei City 11031, Taiwan; 4 Taipei Heart Institute, Taipei Medical University, Taipei City 110301, Taiwan

**Keywords:** acute coronary syndrome, angina, chronic coronary syndrome, coronary artery spasm, coronary microvascular dysfunction

## Abstract

Coronary artery spasm (CAS) is a common endotype in patients with angina with non-obstructive coronary arteries. Pathophysiologically, the presence of CAS among arteries is not normal, as evidenced by several interacting mechanisms involving CAS development, including the endothelium, vascular smooth muscle cells, adventitia, autonomic nervous system, local inflammation, and systemic inflammation. Clinically, CAS is a dynamic process with a threshold effect on presentation; it can present as silent ischemia, atypical chest pain, resting angina, chronic coronary syndrome, acute coronary syndrome, variant angina, and even sudden cardiac arrest. Incomplete intracoronary provocation testing to exclude CAS as the etiology of chronic or acute coronary syndrome leads to an incorrect diagnosis and, subsequently, inappropriate treatment. Identification of the correct endotypes of chronic and acute coronary syndromes is critical for the selection of appropriate therapy, which thus affects disease outcome. Therefore, it is essential to complete intracoronary provocation testing for both the right and left coronary arteries to reach a correct diagnosis regarding CAS, including epicardial vasospasm and microvascular spasm. If CAS is found not to be the cause of myocardial ischemia, then a microvascular functional assessment is the next step to identify the etiology of the ischemic event. A comprehensive assessment of CAS is essential before appropriate treatments can be started.

## 1. Introduction

Coronary artery spasm (CAS), a disease involving vasoconstriction of the epicardial coronary artery (epicardial vasospasm, EVS) and/or coronary microcirculation (microvascular spasm, MVS), results in decreased flow to the myocardium with endocardial and myocardial ischemia [1]. CAS occurs not only in atherosclerotic coronary artery disease [2] but also in angiographically normal coronary arteries [3]. For a long time, CAS was underestimated in daily clinical practice due to a misunderstanding of the associated electrocardiographic changes, an underestimation of the clinical importance of CAS, a larger focus on coronary intervention, and the wide use of calcium antagonists. Apart from a few catheterization laboratories, most interventional cardiologists worldwide have abstained from using provocation testing to identify the etiologies of angiographically normal coronary arteries in patients with chronic coronary syndrome (CCS) and acute coronary syndrome (ACS). The present review provides an overview of the re-emergence of provocative testing for CAS, exhibiting its scientific novelty.

## 2. Methods

The present narrative review summarizes recent progress regarding the pathophysiology and diagnosis of CAS and the importance of verifying CAS as the underlying etiology of CCS and ACS. A search of PubMed/MEDLINE was conducted using the terms “coronary spasm”, “coronary artery spasm”, “coronary microvascular spasm”, and “vasospastic angina” to include English publications from 1999 to 2025. A total of 2267 articles, including original articles, randomized clinical trials, meta-analyses, guidelines, and systematic reviews, were screened. The publications that contained important and/or novel information were included in this practice-based review.

## 3. Understanding Coronary Artery Spasm

In 1999, the American College of Cardiology/American Heart Association published guidelines for coronary angiography [4], stating that intracoronary methylergonovine provocation of CAS is safe, sensitive, and specific. However, at that time, cardiologists were only interested in patients needing angioplasty and stenting. Some may have misunderstood CAS to be a rare disease and viewed provocation testing as time-consuming. Some remained concerned about the safety of intracoronary provocation testing, although it had been declared safe. Recently, investigators have reconfirmed CAS intracoronary provocation testing as a relatively safe procedure and have noted that CAS is not a rare endotype in patients with angina and non-obstructive coronary arteries (ANOCAs) [5,6,7]. Additionally, many investigators have determined that CAS plays an important role in the setting of ACS, out-of-hospital cardiac arrest, myocarditis, hypertrophic cardiomyopathy, and Takotsubo syndrome [8,9,10,11,12]. In 2023, a Japanese guideline on vasospastic angina and coronary microvascular dysfunction emphasized the role of CAS in myocardial infarction with non-obstructive coronary arteries and ischemia with non-obstructive coronary arteries (INOCAs) [13]. In 2024, the European Society of Cardiology published a chronic coronary syndrome guideline which upgraded invasive coronary function testing to a class IB indication for patients with ANOCAs to identify the underlying etiologies of angina [14]. Therefore, we should continue to consider CAS as a possible etiology of CCS or ACS and attempt to confirm the diagnosis of CAS to appropriately manage ischemic symptoms. There is consensus that determining the etiology of patients suffering from CCS and ACS is a necessary initial step to establish personalized healthcare. Recent investigations have noted that CAS is a common endotype in patients with ANOCAs [5,6,7,15,16,17]. Different endotypes can also coexist in a patient. Meanwhile, pure coronary microvascular dysfunction without EVS or MVS is less common in patients with ANOCAs [5,6,7]. Given the above observations, we should familiarize ourselves with the pathology, diagnosis, and treatment of CAS.

Pathophysiologically, an artery with CAS is not normal. CAS without coronary artery disease has been documented in 70% of endomyocardial biopsy-proven PVB19 myocarditis cases [18]. In patients with CAS, intimal injury and neointimal hyperplasia with infiltrating inflammatory cells in coronary plaques or arteries have been documented through autopsy and histological examination [19]. Using intracoronary ultrasound, diffuse intimal thickening was found in the coronary arteries of CAS [20]. A Japanese study found that inflammatory changes in coronary adventitia and perivascular adipose tissue were associated with CAS in vasospastic angina patients, as demonstrated via ^18^F-fluorodeoxyglucose positron emission tomography/computed tomography [21]. Therefore, local inflammation either inside or outside the coronary artery could result in impaired vasodilatation and possibly enhanced vasoconstriction. In addition, ^18^F-fluorodeoxyglucose uptake decreased after treatment with calcium antagonists in the aforementioned CAS patients.

In contrast, systemic inflammation is present in CAS patients [12], as demonstrated by elevated circulatory inflammatory and adhesion markers, peripheral leukocyte Rho-associated coiled-coil-containing protein kinase activity, and disease–entity association such as incident diabetes, Kounis syndrome, bronchial asthma, and anxiety/depression; it is defined as a clinical risk factor associated with cigarette smoking. In our prior study [22], patients in the CAS group were more likely to be smokers and have higher peripheral white blood cell and monocyte counts and higher high-sensitivity C-reactive protein, interleukin-6, soluble intercellular adhesion molecule-1, and soluble vascular adhesion molecule-1 levels. Further analysis showed that interleukin-6 was independently associated with the diagnosis of CAS. Furthermore, we also noted that there was a different association with high-sensitivity C-reactive protein between genders. Diabetes mellitus contributes to CAS development in men with low high-sensitivity C-reactive protein levels, a trend not observed in women. In addition, we found that diabetes mellitus and hypertension are negatively associated with CAS development in patients with high-sensitivity C-reactive protein levels, especially in women. The negative association of hypertension with CAS in that study further suggests that CAS is pathogenically different from coronary atherosclerosis. Our prior preclinical study found that peripheral leukocyte Rho-associated coiled-coil-containing protein kinase activity independently predicted CAS development in patients with ANOCAs [23]. Rho-associated coiled-coil-containing protein kinase is a serine/threonine protein kinase that mediates Rho, a downstream signal, on the actin cytoskeleton, which is activated in patients with CAS. Interestingly, elevated circulatory inflammatory markers, peri-coronary adipose tissue inflammation, and peripheral leukocyte Rho-associated coiled-coil-containing protein kinase activity were reduced after treatment for CAS [23,24,25]. Based on the results of the above studies and other studies [10,21,26,27,28,29], we conclude that local inflammation and systemic inflammation are present in coronary arteries with CAS, suggesting that coronary arteries with CAS are not normal, despite appearing as angiographically normal coronary arteries.

**Figure 1:**
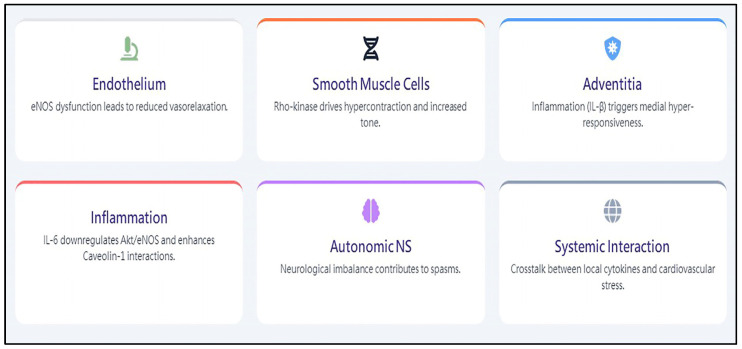
Multi-factorial integration mechanisms of coronary artery spasm.

From the perspective of molecular biomedicine, the cellular mechanisms of CAS development are not well understood. Endothelial nitric oxide synthase is one of the most important sources of nitric oxide production in vasorelaxation [30]. In human endothelial cells, serine/threonine protein kinase B mediated the activation of endothelial nitric oxide synthase, leading to increased nitric oxide production [31]. Endothelial nitric oxide synthase is reversely associated with protein kinase, implicated in opposing regulatory effects on the enzyme, leading to both endothelial nitric oxide synthase activation (namely, serine/threonine protein kinase B) and inhibition (namely, extracellular signal-regulated kinase). Our study in human umbilical vein endothelial cells found that interleukin-6 decreased nitric oxide production and endothelial nitric oxide synthase phosphorylation at Ser1177 through downregulation of serine/threonine protein kinase B phosphorylation, without affecting endothelial nitric oxide synthase protein expression [32]. In addition, interleukin-6 increased the protein–protein interaction between endothelial nitric oxide synthase and caveolin-1, a negative regulator of endothelial nitric oxide synthase, through upregulation of caveolin-1 protein, and increased its half-life. We further demonstrated that the effects of interleukin-6 on the activation of endothelial nitric oxide synthase and the expression of protein–protein interactions between endothelial nitric oxide synthase and caveolin-1 are mediated by the signal pathway of extracellular signal-regulated kinase. Recently, some animal studies determined that sustained adventitial inflammation by interleukin-a beta or drug-eluting stent implantation leads to medial vascular smooth muscle hypercontraction and enhanced coronary contraction through the activation of Rho-kinase [33,34,35]. The expression of Rho-kinase mRNA and RhoA mRNA was increased in the segment of the spastic coronary artery as compared with the segment of the control coronary artery [33]. Furthermore, the inhibition of serotonin-mediated vascular smooth muscle hypercontraction was noted using the Rho-kinase inhibitor Y-27632 [36]. Therefore, cellular mechanisms either inside or outside the vessel wall could lead to vascular smooth muscle hypercontraction, a clinical scenario of CAS. Over the years, many basic studies have tried to elucidate the mechanisms of CAS. However, no single mechanism explains CAS. It is reasonable to speculate that multiple mechanisms involving the endothelium, vascular smooth muscle cells, adventitia, autonomic nervous system, local inflammation, and systemic inflammation are part of CAS development (Figure 1).

Clinically, CAS is a dynamic process with a threshold effect on presentation [37]. CAS can present as silent ischemia, atypical chest pain, resting angina, CCS, ACS, variant angina, and even sudden cardiac arrest [9,37,38,39,40]. Silent ischemia with transient ST segment elevation can be observed on a 24 h Holter electrocardiographic recording [12]. The frequency of a positive CAS provocation test result for patients presenting with atypical chest pain was 1.5% [37]. In contrast, the frequency of provoked CAS in patients with resting angina was 50.4% [37]. CAS was also noted in CCS patients presenting with ANOCAs and positive non-invasive stress test results, i.e., INOCAs [41]. The incidence of positive CAS provocation in INOCA patients was 67% [41], indicating that CAS is common in INOCAs. The identification of CAS has become more important in the present primary coronary intervention era, given the range of treatment strategies available for atherosclerotic coronary artery disease and CAS. In 25% of patients with ACS, no significant coronary artery disease was found, and CAS could be provoked in about half of these patients [8,40]. According to our prior report, 12% of patients with acute myocardial infarction had a patent culprit coronary artery, and in 95% of these patients, infarct-related CAS could be provoked [12]. Moreover, CAS is a cause of out-of-hospital cardiac arrest [9], which has been outlined as an important diagnostic issue in a recent guideline from the European Society of Cardiology for the management for survivors of sudden cardiac death [42].

Using the National Health Insurance Research Database, we found that bronchial asthma was independently associated with newly developed vasospastic angina with an odds ratio of 1.85 [43]. In addition, the prevalence, 4.4%, of asthma in vasospastic angina patients was higher than the prevalence of asthma (2.6%) in atherosclerotic coronary artery disease. This finding provides evidence of the interplay between allergic reaction and CAS, which is similar to the observation of Kounis syndrome [44]. Kounis syndrome has three variants: type I, allergic CAS due to endothelial dysfunction in patients without coronary artery disease; type II, CAS or plaque erosion without coronary artery disease due to an allergic reaction; type III, allergic CAS in the presence of coronary thrombosis. We previously reported type I Kounis syndrome in a 45-year-old sigmoid cancer patient who had CAS-related vasospastic angina secondary to an allergy to the chemotherapy agent oxaliplatin [45]. Although an allergic reaction exists in both bronchial asthma and Kounis syndrome, the treatment strategies for Kounis syndrome are quite different. Therefore, knowledge of individual hypersensitivity is essential to manage CAS patients appropriately.

**Figure 2:**
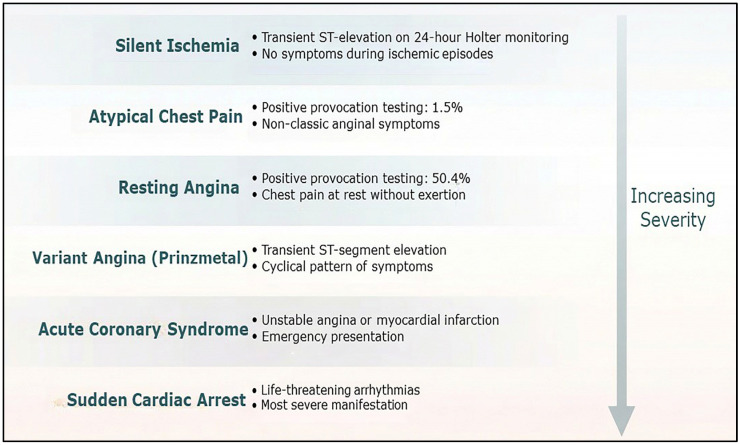
Clinical manifestation of coronary artery spasm.

Aside from the above observation of the clinical cardiac manifestation of CAS, electrocardiographic findings provide additional clues to the occurrence of CAS. The most common electrocardiographic change associated with CAS is ST segment depression and T-wave inversion instead of ST segment elevation, indicating that non-transmural myocardial ischemia occurs in the majority of CAS-induced vasospastic angina cases [46]. This further explains that variant angina, i.e., angina accompanied by transient ST segment elevation, is a manifestation of CAS-induced vasospastic angina [37]. This suggests that there is an unmet need to clinically verify CAS as the cause of a cardiac ischemic event (Figure 2).

## 4. Diagnosis and Treatment of CAS in ACS

Careful interpretation of the 12-lead electrocardiogram in the emergency room plays a key role in the initial management of patients with acute onset of chest pain. New ST segment elevation on electrocardiogram does not definitely indicate acute transmural myocardial injury. Some diseases other than ACS may present with new ST segment elevation in the emergency room. ACS mimickers include subarachnoid hemorrhage, acute pericarditis, and Takotsubo syndrome. Therefore, follow-up electrocardiograms in conjunction with the clinical presentation represent the key factors in reaching a correct diagnosis. If electrocardiographic changes resolve after nitroglycerin is given, a diagnosis of CAS-induced ACS can be made. If electrocardiographic changes persist, an emergency coronary intervention might be necessary. Meanwhile, it is very important to diagnosis subsequent new electrocardiographic ST segment changes, as they could be associated with atherosclerotic coronary artery stenosis, CAS, coronary artery dissection, plaque rupture/erosion, or acute myocarditis; such changes determine the subsequent management strategies. After careful interpretation of coronary angiography and/or intracoronary ultrasound imaging to exclude other etiologies of ACS, especially coronary artery dissection or plaque rupture/erosions, subsequent EVS and MVS provocation testing is the next step to make the diagnosis of CAS-induced ACS.

**Figure 3:**
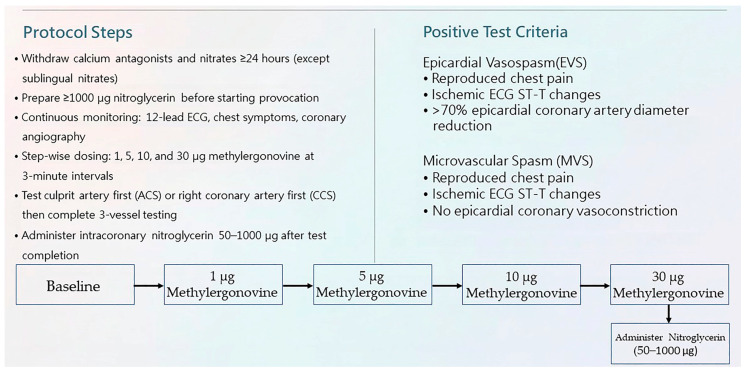
Intracoronary provocation testing protocol and interpretation.

CAS includes EVS and MVS. Final confirmation is achieved via intracoronary provocation testing using two agents, ergonovine and acetylcholine [13,14]. Methylergonovine, an analog of ergonovine, is an alternative agent for provoking EVS with similar sensitivity, specificity, and safety [4]. Both agents induce endothelium-dependent vasodilation in the presence of nitric oxide released from a normal endothelium [47]. In contrast, in the presence of an abnormal endothelium, vasoconstriction occurs due to inadequate nitric oxide production [48]. We have used intracoronary methylergonovine provocation testing to identify CAS since 1998 [40], with the protocol as follows (Figure 3):

(1) Withdraw calcium antagonists and nitrates except sublingual nitrates for at least 24 h;
(2) Prepare at least 1000 μg nitroglycerin before starting provocation;
(3) Continuously monitor the 12-lead electrocardiogram, patient chest symptoms, and coronary angiography;
(4) Administer methylergonovine at 3 min intervals with step-wise dosing of 1, 5, 10, and 30 µg, first into the ACS culprit artery and subsequently into the remaining arteries;
(5) Administer intracoronary nitroglycerin 50–1000 µg at the end of provocation of the right or left coronary artery, depending on the hemodynamic status irrespective of the test result.

**Figure 4:**
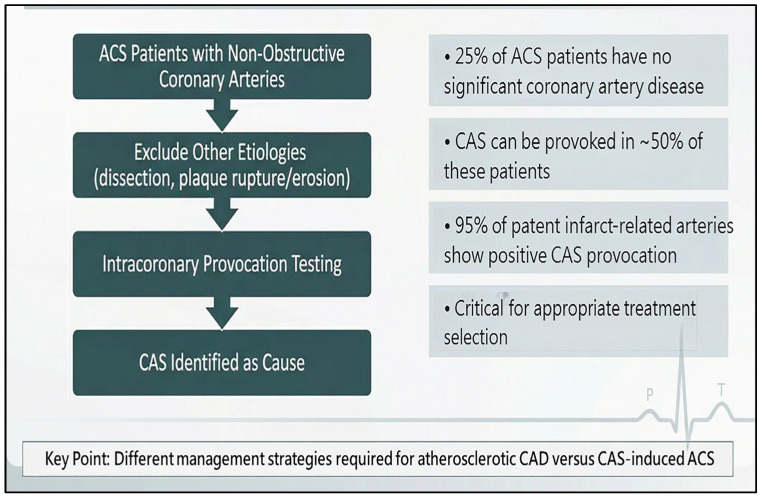
Role of provocation testing in acute coronary syndrome.

Once the diagnosis is confirmed, the provocation procedure is stopped. A positive provocation test result for EVS includes reproduced chest pain, ischemic electrocardiographic ST-T changes, and an epicardial coronary artery diameter reduction of more than 70% compared with the vessel diameter after intracoronary nitroglycerin administration [41]. MVS is defined as reproduced chest pain, ischemic electrocardiographic ST-T changes, and no epicardial coronary artery vasoconstriction [13]. The spasm provocation protocol does not require a flow/pressure wire, which is required for the subsequent coronary microvascular function assessment. In theory, the coronary arteries and microvasculature should be fully dilated to assess their resistance, which is an important parameter in assessing coronary microvascular function. Invasive coronary microvascular dysfunction is commonly defined by preserved epicardial flow in conjunction with impaired microvascular indices, which includes a reduced coronary flow reserve ≤2.5 and/or an elevated index of microvascular resistance >25 [49]. Therefore, after intracoronary nitroglycerin administration (Figure 4), we perform a complete coronary function assessment, which first includes CAS provocation testing followed by coronary microvascular function assessment, which is consistent the CCS guideline developed in 2024 by the European Society of Cardiology [14].

Treatments for CAS include non-pharmacological and pharmacological strategies. A non-pharmacological strategy is the cessation of cigarette smoking because it is a risk factor for CAS development [12]. Pharmacological strategies include prescription of a first-line agent such as calcium antagonists [13,14] and the avoidance of non-selective beta-blockers. It is suggested that calcium antagonists should be long-acting and used before going to bed, with a larger dose than that used in treating hypertensive patients, e.g., diltiazem usually given at 240–360 mg/day. One important point is that an oral long-acting calcium antagonist should be given shortly after CAS provocation, because CAS may recur during that night. This is especially important when caring for ACS patients, because their vasomotion disorder is of high disease activity. Nitrate can resolve CAS quickly; however, its efficacy is limited by intolerance and poor long-term clinical outcomes [50,51,52]. Coronary intervention for CAS-induced ACS is contraindicated, especially in patients with angiographically normal coronary arteries, because of diffuse and high spastic disease activity in the setting of CAS-induced ACS [13,14,53,54,55].

## 5. Diagnosis and Treatment of CAS in CCS

**Figure 5:**
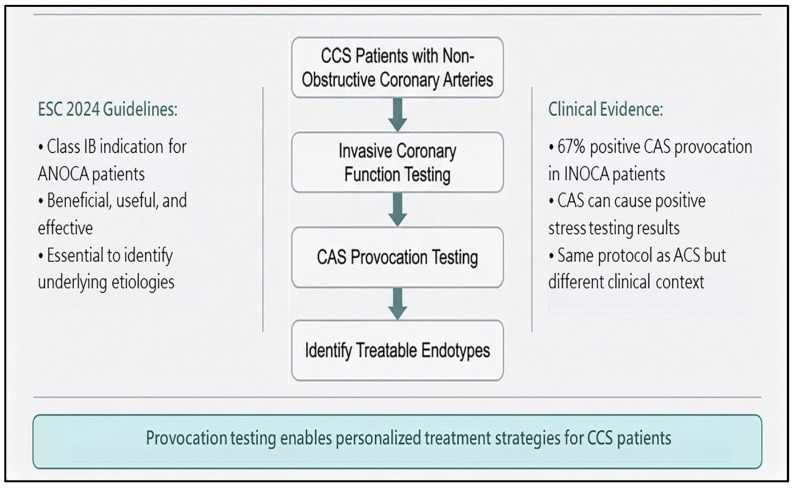
Role of provocation testing in chronic coronary syndrome.

CAS is a manifestation of CCS. Impaired myocardial flow is the pathogenic mechanism for CCS. Patients with CAS may present with atypical chest pain, thus preventing its early diagnosis and appropriate treatment. CAS can cause reduced coronary blood flow during non-invasive stress testing with a resultant non-conclusive or positive stress test result [41]. Our prior study showed that a positive non-invasive stress test result could be obtained in 67% of CAS patients with INOCAs [41]. Regardless, a definite diagnosis of CAS depends on invasive CAS provocation testing. The CAS provocation protocol for CCS is the same as that for ACS (Figure 5). In patients with CCS, we usually perform CAS provocation testing for the right coronary artery first and then for the left coronary artery. Treatment strategies for CAS in patients with CCS are almost the same as for CCS. Recently, we developed a formula to predict CAS using clinical and echocardiographic parameters [56] to avoid missing the diagnosis of vasospastic angina during daily practice.

Pharmacological treatment with calcium antagonists is suggested to be lifelong due to the persistent spasticity of coronary arteries and the likelihood of silent myocardial ischemia [12]. Silent myocardial ischemia could be fatal due to myocardial ischemia-related lethal cardiac arrhythmias and sudden cardiac death. A combination of two different calcium antagonist mechanisms is suggested for recurrent CAS [12]. Some other pharmacological therapies are also beneficial in treating CAS. Rho-associated coiled-coil-containing protein kinase inhibitors [57,58] and statins [59,60] are found to be beneficial in treating CAS. Since EVS, MVS, and coronary microvascular dysfunction frequently coexist in patients with ANOCAs, ranolazine, a late inward sodium current inhibitor, can be used as an additive agent for patients with recurrent angina after administering optimal doses of a calcium antagonist [61]. Coronary intervention is not an appropriate option to treat CAS in the absence of adequate doses of calcium antagonists and the cessation of cigarette smoking [13,14]. Non-pharmacological treatment, namely the cessation of smoking, and pharmacological treatments, including calcium antagonists, nitrates, and ranolazine, are the primary elements of CAS treatment.

## 6. Safety and Feasibility of CAS Provocation Testing

There is consensus that intracoronary CAS provocation testing has an acceptable low rate of complications [62]. Our prior study and a Japanese multicenter study reported a complication rate of 0.66–0.8% during intracoronary ergonovine provocation testing [62,63]. The rate of serious cardiac arrhythmias is higher with intracoronary acetylcholine than with ergonovine testing. Intracoronary CAS provocation testing has been supported by prior studies in the setting of ACS [8,40,42,64,65]. A misdiagnosis or delayed diagnosis of CAS-induced ACS may lead to subsequent inappropriate treatment. Therefore, intracoronary CAS provocation in ACS can be recommended after excluding atherosclerotic causes of ACS.

For CCS patients with ANOCAs or INOCAs, CAS provocation testing was recommended in 2024 by the European Society of Cardiology as a Class IB indication to identify the treatable endotypes [14]. In other words, CAS provocation testing is beneficial, useful, and effective in patients with ANOCAs or INOCAs. Furthermore, methylergonovine or acetylcholine provocation testing was recommended in 1999 by the American College of Cardiology/American Heart Association/Society for Cardiac Angiography and Interventions to identify CAS, supported by its safety, sensitivity, and specificity [4]. Nevertheless, we must also recognize the contraindications for intracoronary CAS provocation testing, namely pregnancy, severe hypertension (systolic blood pressure > 180 mm Hg), moderate-to-severe aortic stenosis, and uncontrolled ventricular arrhythmia [4].

## 7. Main Key Points

(1) Perform complete intracoronary provocation testing for both right and left coronary arteries in the absence of contraindications.
(2) Do not abandon attempts to confirm CAS diagnosis.
(3) Provide appropriate anti-spastic treatment with calcium antagonists.
(4) Coronary intervention for CAS-induced ACS and CCS is contraindicated, especially in patients with angiographically normal coronary arteries.

## 8. Conclusions

**Figure 6:**
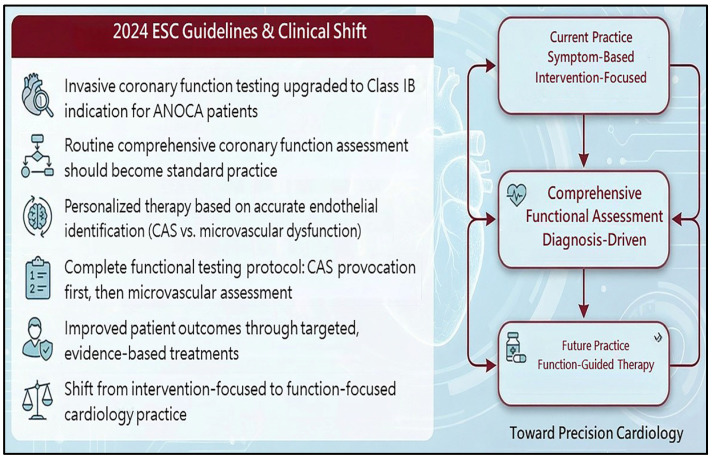
Future directions and clinical implications of coronary artery spasm.

The correct diagnosis of ANOCAs and INOCAs through complete CAS provocation testing of three coronary arteries and coronary microvascular function assessment is fundamental to effective and life-saving treatment. Preventing the recurrence of vasospastic angina is the best strategy for treating CAS-induced CCS or ACS, and it is our obligation and responsibility to help these patients, because, with or without recurrence, CAS is not a benign condition and can be fatal. Addressing CAS following a correct diagnosis is urgent because appropriate treatment can only be determined on this basis (Figure 6).

## 9. Future Directions

There will always be CAS patients. Many randomized controlled trials have demonstrated the importance of identifying the CAS-induced clinical ischemic event. Future cardiology guidelines will enhance evidence-based medicine for the diagnosis and treatment of CAS. Therefore, the era of precision cardiology is coming.

The underlying pathophysiology of CAS remains elusive despite many association and intravascular imaging studies. Furthermore, the interplay between the endothelium, smooth muscle, adventitia, inflammation, and CAS-induced vasospastic angina is still unclear and needs further basic and clinical studies. Finally, specific biomarkers, genetic factors, or advanced imaging techniques will provide more information on the cause–effect of CAS development.

## Data Availability

Not applicable.

## Abbreviations

The following abbreviations are used in this manuscript:

CAS	Coronary Artery Spasm
CCS	Chronic Coronary Syndrome
ACS	Acute Coronary Syndrome
ANOCA	Angina and Non-Obstructive Coronary Artery
INOCA	Ischemia with Non-Obstructive Coronary Artery
EVS	Epicardial Vasospasm
MVS	Microvascular Spasm
